# Editorial: The commonality in converged pathways and mechanisms underpinning neurodevelopmental and psychiatric disorders

**DOI:** 10.3389/fnmol.2023.1349631

**Published:** 2023-12-20

**Authors:** Qijian Deng, Ling Hu, Yu-Qiang Ding, Bing Lang

**Affiliations:** ^1^Department of Psychiatry, National Clinical Research Center for Mental Disorders and National Center for Mental Disorders, The Second Xiangya Hospital, Central South University, Changsha, China; ^2^State Key Laboratory of Medical Neurobiology and MOE Frontiers Center for Brain Science, Institutes of Brain Science, Fudan University, Shanghai, China

**Keywords:** neurodevelopment, major depressive disorder, schizophrenia, potential biomarkers, molecular pathways

## Introduction

In the past several decades, enormous scientific achievements have led to increasing appreciation of the relationship between neurodevelopment and psychiatric disorders, a fast-growing research arena in neuropsychiatry. In this topic issue, we have collected seven articles spanning from fundamental cellular and molecular research to clinical study, and aimed to provide the latest advancements as well as future research directions in neuropsychiatry focusing on the developmental biology. Here we outline the contribution and implication for future study of the seven articles in this Research Topic across the following research areas: (1) An updated list of currently reported CCCTC-binding factor (*CTCF*) mutations and potential involvement in neural developmental disorders; (2) A longitudinal epigenomic study featuring molecular pathways actively involved in the therapy response and remission in depressed patients; (3) potential biomarkers for schizophrenia (SCZ) and Alzheimer's disease; (4) Effects of prenatal exposure to synthetic sex hormones on neurodevelopment.

## *CTCF* and neural developmental disorders

*CTCF* is a ubiquitously expressed and highly conserved zinc finger protein. It can function as either a transcription activator, repressor or even insulator protein (to block the communication between enhancers and promoters) and thus remain functionally versatile. It can attract many other transcription factors while bind to chromatin domain boundaries and help the establishment of the three-dimensional organization of the chromatin. Due to the existence of a myriad of *CTCF* binding sites across the whole genome which can actively jump around as retrotransposable sequences, *CTCF* plays profoundly or even unpredicably regulatory roles for the process of genomic transcription. Indeed, CTCF null knockout mice present early embryonic lethality. *CTCF* haploinsufficiency mice display altered DNA methylation landscape and dysregulated gene expression patterns and are predisposed to both spontaneous and induced tumor incidence (Alharbi et al., 2021). Conditional deletion of *CTCF* at various stages of development in mice can cause disorganized brain development, increased apoptosis, behavioral and learning deficits and even premature death (Hirayama et al., 2012; Davis et al., 2022). These data strongly highlight the essential role of CTCF for proper neurodevelopment.

The first pathogenic *CTCF* variant was identified in individuals diagnosed with neurodevelopmental disorder (NDD) phenotypes in 2013 (Gregor et al., 2013). Since then, a total of 76 CTCF variants have been described in over 100 individuals with variable but overlapping phenotypes for NDD and neuropsychiatric disorders, which include but not limited to global developmental delay, intellectual disability, communication disorders, autism spectrum disorders, attention deficit hyperactivity disorder, anxiety and motor disorders. In this Research Topic, Price et al. aimed to utilize the ever-growing exome sequencing datasets derived from published NDD cohorts to uncover novel pathogenic CTCF variants and to produce an updated genotype-phenotype data repository. They performed comprehensive search in 11 publicly accessible databases including ClinVar, DECEPHER, AutDB, Developmental Brain Disorder Gene Database, Denovo-DB, DisGeNET, EGIdb, Gene4denovo, LOVD, SFARI and VariCarta, and found an additional 86 CTCF variants including sequence nucleotide variants and copy number variation. Importantly, the majority of pathogenic CTCF variants identified in association with NDD phenotypes were missense mutations affecting the protein coding sequence and mostly clustered across zinc finger domains. Many of those mutations at key zinc finger residues were predicted to result in loss- or gain-of-function. Hence the analysis by Price et al. not only provide a comprehensive shortlist for all currently known CTCF mutations but is of great value for basic and translational research.

## DNA methylation and major depressive disorder

MDD is a genetic and phenotypic complex disorder with a lifetime prevalence of 15–20%. It is episodic and often accompanied by considerable morbidity, excess mortality, increased risk of suicide and substantial costs. The etiology of MDD remains highly debatable, with both genetic alteration and environmental stressors involved. DNA methylation at cytosine-guanine dinucleotides (CpG) sites is one of the most studied epigenetic markers and there is growing evidence of its role in understanding depression. DNA methylation is influenced by both genetic and environmental factors, that means, it on one hand depends on the underlying genomic makeup, but also alters genetic expressing landscape upon environmental stress. Hence it is in a unique position to investigate changes of molecular signatures at early stage of drug treatment. For this purpose, Assche et al. designed a randomized controlled trial with MDD patients and assessed the DNA methylation of peripheral blood at both baseline and 8 weeks after cognitive intervention. They compared the methylation profile between responders and non-responders, followed by longitudinal analyses within individuals. Interestingly, the most significant difference in CpG methylation comparing response vs. non-response was found within the *IQSEC1* gene, which is highly involved in the control of processes such as endocytosis of plasma membrane proteins, E-cadherin recycling and actin cytoskeleton remodeling. Meanwhile, the most significant GO-terms for the response were linked to telomeres. For longitudinal response analysis, sodium transport pathways were returned for the responders and phosphatase regulation plus synaptic functioning was associated with remission. Although no CpG was found genome-wide significant, these data strongly suggest the value of altered DNA methylation as an early signature to capture the therapy response or remission in MDD patients and should be warranted for further investigation.

## Neurodevelopment, SCZ, and Alzheimer's disease

SCZ is a chronic, debilitating disease with strong origin of neurodevelopment. Similar with MDD, both genetic and environmental factors contribute to the etiology of the disease and the heritability is estimated as high as 60–80%. Despite the great progress in the past several decades, only a handful of causal genes have been discovered and cross-validated by populations of different ethnicity worldwide. In the current issue, two articles have been collected. Using the method of amplicon targeted sequencing, Yin et al. have discovered 5 novel variants of the *vasoactive intestinal peptide receptor 2* gene (*VIPR2*) through the cohort of 1804 Chinese Han patients and 996 healthy controls. Specifically, the variants rs78564798, rs372544903 and a novel mutation chr7:159034078 (GRCh38) were significantly associated with SCZ, indicating that *VIPR2* may be a strong candidate of susceptibility loci of SCZ. *VIPR2* encodes VPAC2 receptor which can bind neuropeptides VIP and PACAP. Consistently, aberrant expression of VPAC2, VIP or PACAP can profoundly affect neurite outgrowth and extension and the genetically modified mice present impaired learning/memory and cognition reminiscent of human SCZ. However, the values of these variants still need to be cross-validated in different cohorts with larger sample size. SCZ patients have long been proposed to show a longer developmental trajectory. In the 2nd article, Zhang Z. et al. have confirmed a delayed establishment of independent walking for the first episode SCZ patients which was positively associated with low periphery BDNF levels. This is consistent with the idea that SCZ patients have a presumably longer developmental trajectory. Although it is still considerably arguable, most of the literature tends to acknowledge a reduced BDNF level in the brains of SCZ sufferers. Hence it would be appealing to test how the antipsychotic drugs affect BDNF at both periphery and brains through large cohort analysis as this may provide a simple but effective biomarker for early diagnosis and prevention of subsets of SCZ.

Growth-associated protein 43 (GAP-43) is known as a growth and plasticity protein due to the high expressing level in neuronal growth cones during development and axonal regeneration. In this issue, Lee et al. reported that GAP-43 may synergically work with BDNF, a neurotrophin essential for neuronal development and survival, synaptic plasticity, and cognitive function. They further argued that this interaction could be harnessed as a novel therapeutic target of Alzheimer's disease though the physical binding between GAP-43 and BDNF still to be warranted. Zhang Q. et al. have described a special subset of astrocytes expressing both *N-myc* downstream-regulated gene 2 (NDRG2) and nestin upon brain injury. They further showed that this co-localization highly depended on the integrity of Notch signaling pathway.

## Synthetic sex hormones on neurodevelopment

Soyer-Gobillard et al. have reviewed the effects of prenatal exposure to synthetic sex hormone on neurodevelopment, an extremely important but hugely understudied topic. Synthetic sex hormones, namely xenoestrogens and progestins, have been applied to millions of women worldwide to prevent miscarriage or for comfort with great success since the middle of last century and only recently has their effects to neuropsychiatric disorders been scratched. Compared with natural estrogenic hormone, the xenoestrogens (e.g., diethylstilbestrol, DES; or 17-α-ethinyl estradiol, EE) are highly lipophilic which enables them or their metabolites to deposit in the adipose tissue. The long-term genotoxic effects are multifaceted, especially genome hypermethylation which reshapes the expressing profile of many important genes regulating neurodevelopment (for example, *ZFP57* and *ADAMTS9*). These alterations predispose children who have ever been exposed at fetal stage to a set of psychiatric disorders such as SCZ, depression, eating disorders, anxiety and so on. Similarly, progestin can downregulate the expression of ERβ and its target genes through hypermethylation. In addition, its metabolites dihydroprogesterone and allopregnanolone (or iso-pregnanolone) can potently target GABA-A receptor and cause disturbed synaptic plasticity via imbalanced excitatory/inhibitory neural transmission during brain development. This is further supported by the higher risk for the children to develop autism spectrum disorders (ASD).

Apart from the direct influence to the fetus, Soyer-Gobillard et al. (2021) has also raised another important but globally overlooked issue, that is, the potential transgeneration effects of DES. In a case report, they have observed that DES may associate with a range of psychiatric disorders including ASD (boys), bipolar disorder (girls), dyspraxia and learning disabilities, to the children of 3rd or even the 4th generation in family (Gaspari et al., 2021; Soyer-Gobillard et al., 2021). Kioumourtzoglou et al. (2018) also described higher risk of attention deficit/hyperactivity disorder in grandchildren after DES exposure. Although no publication is yet available about the transgenerational influence of progestin, the environmental contamination caused by the ubiquitous presence of synthetic sex hormone is fast-growing. Li et al. (2018) have reported that in China, artificially cultivated fish and shrimp are growing bigger, fatter, and faster, due to the frequent use of estrogen-progestogen contraceptive products. This may play a role to the sharp rise of ASD in Chinese population.

## Conclusion

Mounting data on the genetic research strongly shows that psychiatric diseases lie in the neurodevelopmental continuum. However, despite current advances in the proposed neurodevelopmental hypothesis in the past decades, the converged pathways and mechanisms underpinning neurodevelopmental and psychiatric disorders still remain far from clear. Studies collected in this Research Topic strongly show that a better integrating existing knowledge that allows people to predict the risk of developing neuropsychiatric disorders and concomitant brain alterations that occur throughout the whole developing stages will bring new insights to understand the commonality of the two fields. This will also help to uncover novel biomarkers and to develop new therapy strategies, at least for subsets of psychiatric patients. However, as the whole-exome or genome sequencing is producing more and more larger datasets, an unprecedentedly complexed genetic landscape for psychiatric disorders has just been appreciated and the rodents-based animal research may not fully capture the full genetic complexity of human patients. We predict that the alternatively novel approaches such as induced pluripotent stem cells will be a valuable and promising approach which shall be warranted for the future mechanistic study for neurodevelopmental or neuropsychiatric diseases.

## Author contributions

QD: Conceptualization, Data curation, Methodology, Writing—original draft, Writing—review & editing. LH: Conceptualization, Methodology, Writing—review & editing. Y-QD: Project administration, Supervision, Validation, Writing—review & editing. BL: Formal analysis, Project administration, Supervision, Writing—original draft.
